# Supplementary material for: Empirical methods for controlling false positives and estimating confidence in ChIP-Seq peaks
Source: BMC Bioinformatics. 2008 Dec 5;9:523. doi: 10.1186/1471-2105-9-523 (PMC2628906; doi:10.1186/1471-2105-9-523)
Supplement: Additional File 1 — A variety of html documents from the USeq web site detailing the available applications, their best usage, output file type descriptions, command line menus, etc. [file 1471-2105-9-523-S1.zip › USeqUserGuides/JavaDoc.zip!/JavaDoc/trans/main/PosRegion.html]

PosRegion


|  |  |  |
| --- | --- | --- |
| **Overview** \| **Package** \| **Class** \| **Use** \| **Tree** \| **Deprecated** \| **Index** \| **Help** | |  |
| **PREV CLASS**   **NEXT CLASS** | **FRAMES**    **NO FRAMES**     **All Classes** |  |
| SUMMARY: NESTED \| FIELD \| CONSTR \| METHOD | DETAIL: FIELD \| CONSTR \| METHOD |  |


---


## trans.main Class PosRegion

```
java.lang.Object
  trans.main.PosRegion
```

**All Implemented Interfaces:**: java.lang.Comparable

---

``` public class PosRegion extends java.lang.Object implements java.lang.Comparable ```

For holding information about a genomic interval, what genes are up, down, overlap, or contain this genomic inteval.

---

**Constructor Summary**

`PosRegion(java.lang.String name,
java.lang.String chromosome,
int start,
int end)`


| **Method Summary** | |
| --- | --- |
| `int` | `compareTo(java.lang.Object obj)` |
| `java.lang.String` | `getChromosome()` |
| `Interval` | `getClosestInterval()` |
| `int` | `getClosestNonOverlappingInterval()` |
| `int` | `getEnd()` |
| `java.lang.String` | `getName()` |
| `java.util.ArrayList` | `getOverlapping()` |
| `PosRegion` | `getRegion()` |
| `int` | `getStart()` |
| `void` | `setChromosome(java.lang.String chromosome)` |
| `void` | `setClosestInterval(Interval closestInterval)` |
| `void` | `setClosestNonOverlappingInterval(int closestOverlap)` |
| `void` | `setEnd(int end)` |
| `void` | `setName(java.lang.String name)` |
| `void` | `setOverlapping(java.util.ArrayList overlapping)` |
| `void` | `setRegion(PosRegion region)` |
| `void` | `setStart(int start)` |
| `java.lang.String` | `summaryLine()`             Returns tab delimited line: rank, chrom, start, end, dist to closes gene, gene name(s), # neighbors |

**Methods inherited from class java.lang.Object**

`equals, getClass, hashCode, notify, notifyAll, toString, wait, wait, wait`

**Constructor Detail**

### PosRegion

```
public PosRegion(java.lang.String name,
                 java.lang.String chromosome,
                 int start,
                 int end)
```


**Method Detail**

### compareTo

```
public int compareTo(java.lang.Object obj)
```

:   **Specified by:**: `compareTo` in interface `java.lang.Comparable`

---


### summaryLine

```
public java.lang.String summaryLine()
```

:   Returns tab delimited line: rank, chrom, start, end, dist to closes gene, gene name(s), # neighbors

---


### getChromosome

```
public java.lang.String getChromosome()
```

---


### getStart

```
public int getStart()
```

---


### getEnd

```
public int getEnd()
```

---


### getClosestNonOverlappingInterval

```
public int getClosestNonOverlappingInterval()
```

---


### setClosestNonOverlappingInterval

```
public void setClosestNonOverlappingInterval(int closestOverlap)
```

---


### getName

```
public java.lang.String getName()
```

---


### setName

```
public void setName(java.lang.String name)
```

---


### getOverlapping

```
public java.util.ArrayList getOverlapping()
```

---


### setOverlapping

```
public void setOverlapping(java.util.ArrayList overlapping)
```

---


### getRegion

```
public PosRegion getRegion()
```

---


### setRegion

```
public void setRegion(PosRegion region)
```

---


### setChromosome

```
public void setChromosome(java.lang.String chromosome)
```

---


### setEnd

```
public void setEnd(int end)
```

---


### setStart

```
public void setStart(int start)
```

---


### getClosestInterval

```
public Interval getClosestInterval()
```

---


### setClosestInterval

```
public void setClosestInterval(Interval closestInterval)
```


---


|  |  |  |
| --- | --- | --- |
| **Overview** \| **Package** \| **Class** \| **Use** \| **Tree** \| **Deprecated** \| **Index** \| **Help** | |  |
| **PREV CLASS**   **NEXT CLASS** | **FRAMES**    **NO FRAMES**     **All Classes** |  |
| SUMMARY: NESTED \| FIELD \| CONSTR \| METHOD | DETAIL: FIELD \| CONSTR \| METHOD |  |


---
